# Association Between Antibiotic Receipt and Provider Rating Within a National Telemedicine Practice

**DOI:** 10.1093/ofid/ofae310

**Published:** 2024-06-03

**Authors:** Greeshmasree Kambam, Shiva Salehian, Cindy M Liu, Nina Kleinschmidt, Kristin Dean, Vibin Roy, Jaclyn Marshall, Rana F Hamdy

**Affiliations:** Department of Epidemiology, George Washington Milken Institute of Public Health, Washington, DC, USA; Department of Health Policy, Virginia Commonwealth University, School of Medicine, Richmond, Virginia, USA; Division of Infectious Diseases, Children's National Hospital, Washington DC, USA; Antibiotic Resistance Action Center, Department of Environmental and Occupational Health, George Washington Milken Institute of Public Health, Washington, DC, USA; Department of Clinical Care and Research, Included Health, San Francisco, California, USA; Department of Clinical Care and Research, Included Health, San Francisco, California, USA; Department of Clinical Care and Research, Included Health, San Francisco, California, USA; Department of Clinical Care and Research, Included Health, San Francisco, California, USA; Division of Infectious Diseases, Children's National Hospital, Washington DC, USA; Antibiotic Resistance Action Center, Department of Environmental and Occupational Health, George Washington Milken Institute of Public Health, Washington, DC, USA; Department of Pediatrics, George Washington University, School of Medicine and Health Sciences, Washington, DC, USA

**Keywords:** antibiotic prescriptions, antibiotic stewardship, patient satisfaction, provider ratings, telemedicine, upper respiratory illnesses

## Abstract

This retrospective cohort study estimated the association between prescription receipt and provider 5-star rating for adult visits with upper respiratory infections in a national telemedicine practice with active antibiotic stewardship initiatives. The odds of a 5-star rating were higher for visits with an antibiotic or nonantibiotic prescription and longer visits.

Acute respiratory tract infections (RTIs) are one of the most frequent indications for telehealth consultations in this rapidly-expanding health care setting [1, 2]. The vast majority of acute RTIs are caused by viruses, for which antibiotics are not indicated. Nevertheless, antibiotics are frequently prescribed and can lead to antibiotic resistance and other harmful side effects [3]. Reasons for antibiotic overprescribing for acute RTIs (most notably upper respiratory infections [URIs] and bronchitis) include provider assumptions about patients’ expectations and visit time constraints [4]. Provider concerns of receiving a poor patient rating if antibiotics are not prescribed has also been reported in provider surveys as a contributing factor to antibiotic overprescribing [4]. Patient-reported provider ratings are easily obtainable metrics frequently used in the United States to rank health care systems [5], determine reimbursement [6], and are incorporated into provider financial incentives [7]. However, provider ratings are not a measure of quality of care and in some instances may be at odds with quality measures [8]. Previous analyses have shown an association between higher patient satisfaction and antibiotic prescribing among telemedicine visits for adults with acute RTIs [9]. The study's objective was to evaluate the association between antibiotic prescription receipt and provider ratings among adult patients with symptoms of acute RTIs and to determine the modifying effect of visit duration in a national virtual practice with active antibiotic stewardship initiatives. We hypothesized that by training clinicians on patient communication regarding antibiotics, the association would be smaller.

## METHODS

This retrospective cohort study included visits for patients aged 18 years or older with a URI or bronchitis diagnosis between May 2021 and April 2022 at a national virtual practice, Included Health, where providers solely deliver medical care through video visits. Providers completed antibiotic stewardship training during onboarding and had access to antibiotic stewardship clinical guidelines and ongoing training through Continuing Medical Education lectures. The onboarding training included an online webinar offered by Stanford Medical School that reviewed appropriate use of antimicrobials in the outpatient setting and demonstrated effective patient communication approaches via role playing [10].

Visits for URI or bronchitis were identified based on the *International Classification of Diseases, Tenth Edition* (see Supplementary Table 1). Visits were excluded if: duration was less than 1 or greater than 150 minutes (because these were likely erroneous), a concurrent diagnosis for which antibiotics may be or are indicated or a comorbid condition diagnosis was present at the visit (see Supplementary Methods), or no rating was submitted.

The outcome was provider rating on a 1 to 5 scale in response to “please rate your provider for this visit” immediately after the visit, within the application. More than 95% of the visits received 5 stars. Therefore, we coded the rating as a dichotomous variable: 5-star versus not. The 2 primary independent variables were patient receipt of prescription (antibiotic, nonantibiotic, or none) and visit duration in minutes. Patients who received an antibiotic and nonantibiotic prescription were categorized as receiving an antibiotic. Covariates included characteristics of the visit (primary diagnosis, calendar season), patient (age, gender, geographic location), and provider (type, sex, geographic location, and years in practice).

Our main model was a multivariate logistic generalized estimating equation with provider clustering and robust standard errors. To test whether the relationship between duration and 5-star rating varied by prescription receipt, we ran a second model with an interaction term between the 2 primary variables. We calculated the average marginal effect of 1 additional minute on the probability of a 5-star rating separately by prescription receipt category. Two-sided *P* < .05 was considered statistically significant. We used STATA version 13.0 for analyses.

Children's National institutional review board determined this study was not human subjects research and therefore exempt from institutional review board approval in accordance with 45 CFR §46.102.

## RESULTS

### Visit Characteristics

There were 68 607 visits among 298 providers (median visit volume of 172) during the study period, of which 47 695 (69.5%) had a provider rating. Visits with provider ratings were more likely to be in the spring (20.9% vs 19.8%, *P* = .005) for younger (mean age, 37.1 vs 39.7 years, *P* < .001) and male (39.8% vs 36.6%, *P* < .001) patients, include an antibiotic and nonantibiotic prescription (14.8% vs 14.2% and 62.2% vs 58.3%, *P* < .001, respectively), include a bronchitis diagnosis (24.6% vs 22.5%, *P* < .001), and completed by providers with less time in practice (14.1 vs 14.2 years, *P* < .005) (see Supplementary Table 2).

More than 95% of visits had a 5-star provider rating. Approximately 14.8% of visits (30.2% of bronchitis and 9.8% of URI) included an antibiotic prescription and 62.2% (65% of bronchitis and 62% of URI) had a nonantibiotic prescription. The average visit duration was 9.2 minutes (standard deviation = 4.5) and the average patient age was 37.1 years (standard deviation = 13.9). Approximately 75% of the visits were for URI, and more than half of the patients and providers were located in the South Atlantic and West South Central regions of the United States (Table 1).

**Table 1.** Virtual Visit Characteristics Among Adult Patients With a Diagnosis of Upper Respiratory Infection or Bronchitis, Overall and by Prescription Receipt, May 2021–April 2022

	Visits, No. (%)				
Characteristic	Total (N = 47 695)	Antibiotic Prescription (N = 7074)	Nonantibiotic Prescription (N = 29 654)	No Prescription (N = 10 967)	*P*
Prescription receipt	…	…	…	…	n/a
No prescription	10 967 (23.0)	n/a	n/a	n/a	
Antibiotic	7074 (14.8)	n/a	n/a	n/a	
Nonantibiotic	29 654 (62.2)	n/a	n/a	n/a	
Provider rating	…	…	…	…	<.000
1 star	396 (0.8)	10 (0.1)	121 (0.4)	265 (2.4)	
2 stars	111 (0.2)	4 (0.1)	51 (0.2)	56 (0.5)	
3 stars	334 (0.7)	29 (0.4)	189 (0.6)	116 (1.1)	
4 stars	1475 (3.1)	123 (1.7)	960 (3.2)	392 (3.6)	
5 stars	45 379 (95.1)	6908 (97.7)	28 333 (95.6)	10 138 (92.4)	
Visit duration, mean (SD), min	9.2 (4.5)	9.3 (4.9)	9.4 (4.4)	8.4 (4.4)	
Primary diagnosis	…	…	…	…	<.001
Bronchitis	11 733 (24.6)	3551 (50.2)	7680 (25.9)	515 (4.7)	
Upper respiratory infection	35 962 (75.4)	3523 (49.8)	22 003 (74.2)	10 452 (95.3)	
Season	…	…	…	…	<.018
Spring	9973 (20.9)	1370 (19.4)	6637 (22.4)	1966 (17.9)	
Summer	12 301 (25.8)	1954 (27.6)	7346 (24.8)	3001 (27.4)	
Fall	11 905 (25.0)	1796 (25.4)	7177 (24.2)	2932 (26.7)	
Winter	13 516 (28.3)	1954 (27.6)	8494 (28.6)	3068 (28.0)	
Patient gender	…	…	…	…	<.001
Female	28 669 (60.1)	4428 (62.6)	18 445 (62.2)	5796 (52.9)	
Male	18 964 (39.8)	2639 (37.3)	11 170 (37.7)	5155 (47.0)	
Other	62 (0.1)	7 (0.1)	39 (0.1)	16 (0.2)	
Patient age, mean (SD), y	37.1 (13.9)	39.4 (13.3)	36.8 (13.4)	36.5 (15.3)	<.001
Patient region	…	…	…	…	<.001
East North Central	4174 (8.8)	565 (8.0)	2384 (8.0)	1225 (11.2)	
East South Central	2632 (5.5)	306 (4.3)	1796 (6.1)	530 (4.8)	
Middle Atlantic	2285 (4.8)	366 (5.2)	1284 (4.3)	635 (5.8)	
Mountain	3795 (8.0)	526 (7.4)	2528 (8.5)	741 (6.8)	
New England	605 (1.3)	75 (1.1)	357 (1.2)	173 (1.6)	
Pacific	2924 (6.1)	310 (4.4)	1745 (5.9)	869 (7.9)	
South Atlantic	13 240 (27.8)	2333 (33.0)	7660 (25.8)	3247 (29.6)	
West South Central	14 359 (30.1)	2059 (29.1)	9476 (32.0)	2824 (25.8)	
West North Central	3508 (7.4)	510 (7.2)	2329 (7.9)	669 (6.1)	
Missing	173 (0.4)	24 (0.3)	95 (0.3)	54 (0.5)	
Provider sex	…	…	…	…	.068
Female	32 242 (67.6)	5284 (74.7)	20 016 (67.5)	6953 (63.4)	
Male	15 453 (32.4)	1790 (25.3)	9638 (32.5)	4014 (36.6)	
Provider region	…	…	…	…	.003
East North Central	4633 (9.7)	649 (9.2)	2372 (8.0)	1612 (14.7)	
East South Central	1663 (3.5)	265 (3.8)	976 (3.3)	422 (3.9)	
Middle Atlantic	4218 (8.8)	542 (7.7)	883 (8.4)	1174 (10.7)	
Mountain	4294 (9.0)	883 (12.5)	2586 (8.7)	825 (7.5)	
New England	445 (0.9)	30 (0.4)	237 (0.8)	178 (1.6)	
Pacific	4935 (10.4)	402 (5.7)	3342 (11.3)	1191 (10.9)	
South Atlantic	14 661 (30.7)	2681 (37.9)	8744 (29.5)	3236 (29.5)	
West South Central	10 939 (22.9)	1370 (19.4)	7704 (26.0)	1865 (17.0)	
West North Central	1354 (2.8)	151 (2.1)	849 (2.9)	354 (3.2)	
Missing	553 (1.2)	101 (1.4)	342 (1.2)	110 (1.0)	
Provider years in practice, mean (SD), y	14.1 (9.0)	14.0 (9.0)	13.8 (8.6)	15.2 (9.8)	<.000
Provider type	…	…	…	…	.066
Physician	37 530 (78.7)	5844 (82.6)	22 590 (76.2)	9096 (82.9)	
Nurse practitioner	9660 (20.3)	1183 (16.7)	6747 (22.8)	1730 (15.8)	
Unknown	505 (1.1)	47 (0.7)	317 (1.1)	141 (1.3)	

Abbreviations: n/a, not available; SD, standard deviation.

### Adjusted Odds Ratio of 5-star Provider Rating

Visits with an antibiotic prescription (adjusted odds ratio [AOR], 2.56; 95% confidence interval [CI], 2.03–3.23) or nonantibiotic prescription (AOR, 1.55; 95% CI, 1.34–1.79) had higher odds of a 5-star rating than visits without any prescription. Each additional minute of visit duration increased the odds of receiving a 5-star rating (AOR, 1.03; 95% CI, 1.02–1.05). Other statistically significant covariates associated with higher odds of a 5-star rating were a bronchitis diagnosis, a younger patient, patient residing in the East North Central region, a female provider, and a provider in the East South Central or South Atlantic region (Table 2).

**Table 2.** Unadjusted and Adjusted Odds Ratio of a Visit Receiving a Provider 5-Star Rating

Characteristic	Unadjusted Odds Ratio^a^ N = 47 695 (95% CI)	Adjusted Odds Ratio^b^ N = 47 267 (95% CI)
Prescription receipt	…	…
No prescription	1 [reference]	1 [reference]
Antibiotic prescription	3.40*** (2.55–4.53)	2.56*** (2.03–3.23)
Nonantibiotic prescription	1.75*** (1.44–2.13)	1.55*** (1.34–1.79)
Duration of visit, min	1.04*** (1.02–1.07)	1.03*** (1.02–1.05)
Primary diagnosis	…	…
Bronchitis	1 [reference]	1 [reference]
Upper respiratory infection	0.53*** (0.44–0.63)	0.64***(0.54–0.74)
Season	…	…
Winter	1 [reference]	1 [reference]
Spring	1.09 (0.96–1.25)	1.12 (0.99–1.27)
Summer	1.18 (1.02–1.37)	1.04 (0.92–1.19)
Fall	1.10 (0.96–1.26)	0.92 (0.81–1.06)
Patient gender	…	…
Female	1 [reference]	1 [reference]
Male	0.96 (0.88–1.05)	1.04 (0.94–1.14)
Other	3.02 (0.42–21.74)	2.64 (0.35–19.69)
Patient age, y	0.995** (0.992–0.998)	0.993*** (0.991–0.996)
Patient region	…	…
East North Central	1 [reference]	1 [reference]
East South Central	1.14 (0.86–1.50)	0.92 (0.67–1.26)
Middle Atlantic	1.37 (1.03–1.83)	1.32 (0.98–1.78)
Mountain	1.00 (0.68–1.47)	0.80 (0.59–1.09)
New England	0.98 (0.67–1.44)	0.95 (0.64–1.39)
Pacific	1.06 (0.75–1.52)	1.03 (0.76–1.39)
South Atlantic	1.08 (0.89–1.31)	0.92 (0.72–1.17)
West South Central	1.01 (0.80–1.27)	0.87 (0.66–1.16)
West North Central	0.87 (0.64–1.18)	0.71* (0.52–0.97)
Provider sex	…	…
Female	1 [reference]	1 [reference]
Male	0.66** (0.50–0.89)	0.72** (0.58–0.90)
Provider region	…	…
East North Central	1 [reference]	1 [reference]
East South Central	2.74** (1.44–5.21)	2.65*** (1.59–4.41)
Middle Atlantic	1.32 (0.70–2.49)	1.05 (0.64–1.72)
Mountain	1.76 (0.97–3.19)	1.32 (0.82–2.14)
New England	0.96 (0.40–2.31)	0.74 (0.36–1.53)
Pacific	1.42 (0.76–2.67)	1.25 (0.82–1.93)
South Atlantic	1.90 (1.11–3.26)	1.51* (1.04–2.18)
West South Central	1.64 (0.93–2.93)	1.32 (0.86–2.04)
West North Central	1.29 (0.74–2.22)	1.10 (0.75–1.62)
Provider years in practice, y	0.997 (0.980–1.01)	1.002 (0.99–1.01)
Provider type	…	…
Physician	1 [reference]	1 [reference]
Nurse practitioner	1.001 (0.74–1.35)	0.95 (0.70–1.29)
Unknown	1.02 (0.45–2.32)	1.0001 (0.39–2.56)

Abbreviation: CI, confidence interval.

^a^Unadjusted odds ratio accounts for provider clustering.

^b^Adjusted odds ratio is the output from multivariable logistic generalized estimating equations with provider clustering controlling for all variables presented in the table. STATA removed all observations with any missing values from the multivariable logistic regression (n = 428).

^*^ *P* < .05.

^**^ *P* < .01.

^***^ *P* < .001.

### Marginal Effect of Visit Duration

The predicted marginal effect of one additional minute in visit duration on the probability of a 5-star rating was not statistically significantly different between the 3 groups of prescription receipt (see Supplementary Figure 1).

## DISCUSSION

To our knowledge, this is the first study to examine the relationship between prescription receipt, visit duration, and provider rating within a virtual practice with antibiotic stewardship initiatives. We found a substantially lower rate of antibiotic prescribing, a higher rate of nonantibiotic prescribing (most commonly for symptomatic improvement, including antitussives and nasal sprays), and higher overall provider ratings than previous studies within telehealth practices without similar initiatives [1, 9, 11]. In fact, the antibiotic prescribing rate was almost 75% lower for URI and 50% lower for bronchitis than published rates in telehealth settings [11].

Despite lower antibiotic prescribing rates, a statistically significant relationship between receipt of an antibiotic and nonantibiotic prescription and a 5-star rating remained, similar to the findings of Martinez et al [9]. However, our odds ratios were smaller, which may be driven by the antibiotic stewardship initiatives, which include provider communication training on informing patients why antibiotics are not indicated. Other drivers may be lower patient expectations for a prescription because of only including 2 conditions for which antibiotics are never indicated or by a more recent time period when virtual care is used by a broader patient population. Despite the statistically significant association between prescription receipt and a provider 5-star rating, the high proportion of 5-star ratings among all patient subgroups—92.4% with no prescription, 95.6% with a nonantibiotic prescription, and 97.7% with an antibiotic prescription—bring into question if the differences are meaningful.

In contrast to other studies, we found that a bronchitis diagnosis had almost twice the odds of a 5-star rating than visits with a URI diagnosis. This may be because patients felt validated of their symptoms when given a more “severe” diagnosis. Unlike prior studies [1, 9], we found that a longer visit increased the probability of a 5-star rating. We also tested our hypothesis that a longer visit may be a stronger predictor of a 5-star rating among patients who did not receive an antibiotic than those who did. We found that although the impact of 1 additional minute increased the odds of a 5-star rating for all patients, the magnitude of that effect did not differ significantly by prescription receipt (no prescription, nonantibiotics, and antibiotics).

Our study's strengths include a large sample size of virtual visits in 50 states, delivered by providers who received antibiotic stewardship training. It also has several limitations. First, the large sample size, although a strength, can also lead to statistically significant results that may not be meaningfully different, particularly given that >95% of visits were 5-star ratings. Furthermore, the analysis was performed for those visits (69.5%) for which a provider rating was submitted, which may not be generalizable to all visits. However, the rates of antibiotic receipt were not meaningfully different (14.8% to 14.2%), which increases confidence the findings may be generalizable to visits without rating. Second, limiting the sample to visits with a URI or bronchitis diagnosis excludes visits where antibiotics are not always indicated, such as sinusitis. This precludes our ability to assess for diagnosis shifting, which has been shown to occur with antibiotic stewardship interventions [12]. However, it enabled us to evaluate the relationship of provider rating and receipt of antibiotic, specifically where it is never indicated.

Taken together, our findings support that telemedicine providers can maintain high provider ratings and still adhere to antibiotic stewardship by providing supportive care, such as prescriptions for nonantibiotics, and dedicating more time to explaining treatment choices, including when antibiotics are unnecessary.

## Supplementary Material

ofae310_Supplementary_Data

## Supplementary Data

Supplementary materials are available at *Open Forum Infectious Diseases* online. Consisting of data provided by the authors to benefit the reader, the posted materials are not copyedited and are the sole responsibility of the authors, so questions or comments should be addressed to the corresponding author.

## Notes

***Acknowledgments.*** Drs. Dean, Hamdy, Liu, and Roy, Ms. Kleinschmidt, and Ms. Marshall conceptualized and designed the study. Drs. Kambam and Salehian analyzed the data. Drs. Kambam, Salehian, and Hamdy, and Ms. Marshall drafted the manuscript. All authors critically reviewed the manuscript and take full responsibility for the work. Drs. Kambam and Salehian had full access to all the data in the study and take responsibility for the integrity of the data and the accuracy of the data analysis.

***Data availability.*** Data not publicly available.

***Patient consent statement.*** This study does not include factors necessitating patient consent.

***Financial support*.** This manuscript had no funding source outside of authors’ employment.
