# Supplementary material for: Association Between Antibiotic Receipt and Provider Rating Within a National Telemedicine Practice
Source: Open Forum Infect Dis. 2024 Jun 3;11(7):ofae310. doi: 10.1093/ofid/ofae310 (PMC11234143; doi:10.1093/ofid/ofae310)
Supplement: ofae310_Supplementary_Data [file ofae310_supplementary_data.docx]

**Supplemental Online Content**

**Table 1.** International Classification of Diseases, Tenth Edition (ICD-10) used to Identify Visits for Upper Respiratory Infections and Bronchitis

**Methods.**

**Table 2.** Visits with Provider Ratings compared to Visits without Provider Ratings

**Figure 1.** Average Marginal Effect of One Additional Minute in Visit Duration on the Adjusted Probability of 5-star Rating, by Prescription Receipt

**Table 1. International Classification of Diseases, Tenth Edition (ICD-10) used to Identify Visits for Upper Respiratory Infections and Bronchitis**

| **Condition** | **ICD-10 codes** |
| --- | --- |
| Upper respiratory infection | Diagnosis: J00, J06, J069, J040  Symptoms without diagnosis: R05, R0981, R0982 |
| Bronchitis | J203-J210, J218, J219, J40 |

**Methods.**

The practice includes urgent care, primary care, and behavioral health services. Patients can access the national telehealth practice in a few ways: direct-to-consumer, self-insured employer-provided benefit, or as a virtual provider within their health plan’s network. When registering within the application, patients were prompted to provide information through which their eligibility for services is determined. If they are not eligible through insurance or employer, then they can still access the service as a direct consumer. During this study period, most of the patients accessed the practice through their health plan network.

We excluded visits with a concurrent diagnosis for which antibiotics may be or are indicated or a comorbid condition, such as intestinal infections, urinary tract infections, vasculitis, tuberculosis, thyroid disorders, systemic lupus erythematosus and connective tissue disorders, skin infections, sinusitis, pharyngitis, sickle cell, anemia, sexually transmitted infections including HIV and hepatitis, septicemia, cancers, postprocedural complications, pneumonia, otitis media, other general signs and symptoms, meningitis, arthritis, inflammatory conditions, fungal infections, tonsillitis, bacterial infections, cystic fibrosis, and chronic obstructive pulmonary disease.

**Table 2. Visits with Provider Ratings compared to Visits without Provider Ratings**

| **Characteristic** | **Visits with Provider Ratings N=47,695**  **No. (%)** | **Visits without Provider Ratings N=20,912**  **No. (%)** | **P-value** |
| --- | --- | --- | --- |
| Prescription receipt |  |  | <0.001 |
| No prescription | 10967 (23.0) | 5768 (27.6) |  |
| Antibiotic prescription | 7074 (14.8) | 2962 (14.2) |  |
| Non-antibiotic prescription | 29654 (62.2) | 12182 (58.3) |  |
| Duration of visit, mean (SD), min | 9.2 (4.5) | 9.1 (4.8) | 0.905 |
| Primary diagnosis |  |  | <0.001 |
| Bronchitis | 11733 (24.6) | 4705 (22.5) |  |
| Upper respiratory infection | 35962 (75.4) | 16207 (77.5) |  |
| Season |  |  | <0.005 |
| Spring | 9973 (20.9) | 4132 (19.8) |  |
| Summer | 12301 (25.8) | 5281(25.2) |  |
| Fall | 11905 (25.0) | 5315 (25.4) |  |
| Winter | 13516 (28.3) | 6184 (29.6) |  |
| Patient gender |  |  | <0.001 |
| Female | 28669 (60.1) | 13249 (63.4) |  |
| Male | 18964 (39.8) | 7645 (36.6) |  |
| Other | 62 (0.1) | 18 (0.1) |  |
| Patient age, mean (SD), y | 37.1(13.9) | 39.7(14.3) | <0.001 |
| Patient region |  |  | 0.077 |
| East North Central | 4174 (8.8) | 1884 (9.0) |  |
| East South Central | 2632 (5.5) | 1142 (5.5) |  |
| Middle Atlantic | 2285 (4.8) | 1091 (5.2) |  |
| Mountain | 3795 (8.0) | 1621 (7.8) |  |
| New England | 605 (1.3) | 284 (1.4) |  |
| Pacific | 2924 (6.1) | 1270 (6.1) |  |
| South Atlantic | 13240 (27.8) | 5997 (28.7) |  |
| West South Central | 14359 (30.1) | 5904 (28.2) |  |
| West North Central | 3508 (7.4) | 1638 (7.8) |  |
| Missing | 173 (0.4) | 81 (0.4) |  |
| Provider sex |  |  | 0.132 |
| Female | 32242 (67.6) | 13781 (65.9) |  |
| Male | 15453 (32.4) | 7131 (34.1) |  |
| Provider region |  |  | 0.447 |
| East North Central | 4633 (9.7) | 2294 (11.0) |  |
| East South Central | 1663 (3.5) | 686 (3.3) |  |
| Middle Atlantic | 4218 (8.8) | 1892 (9.1) |  |
| Mountain | 4294 (9.0) | 1936 (9.3) |  |
| New England | 445 (0.9) | 243 (1.2) |  |
| Pacific | 4935 (10.4) | 2192 (10.5) |  |
| South Atlantic | 14661 (30.7) | 6146 (29.4) |  |
| West South Central | 10939 (22.9) | 4678 (22.4) |  |
| West North Central | 1354 (2.8) | 623 (3.0) |  |
| Missing | 553 (1.2) | 222 (1.1) |  |
| Provider years in practice, mean (SD), y | 14.1 (9.0) | 14.2 (9.1) | <0.005 |
| Provider type |  |  | 0.362 |
| Physician | 37530 (78.7) | 16733 (80.0) |  |
| Nurse practitioner | 9660 (20.2) | 3978 (19.0) |  |
| Unknown | 505 (1.1) | 201 (1.0) |  |

**Figure 1. Average Marginal Effect of One Additional Minute in Visit Duration on the Adjusted Probability of 5-star Rating, by Prescription Receipt ^a^**


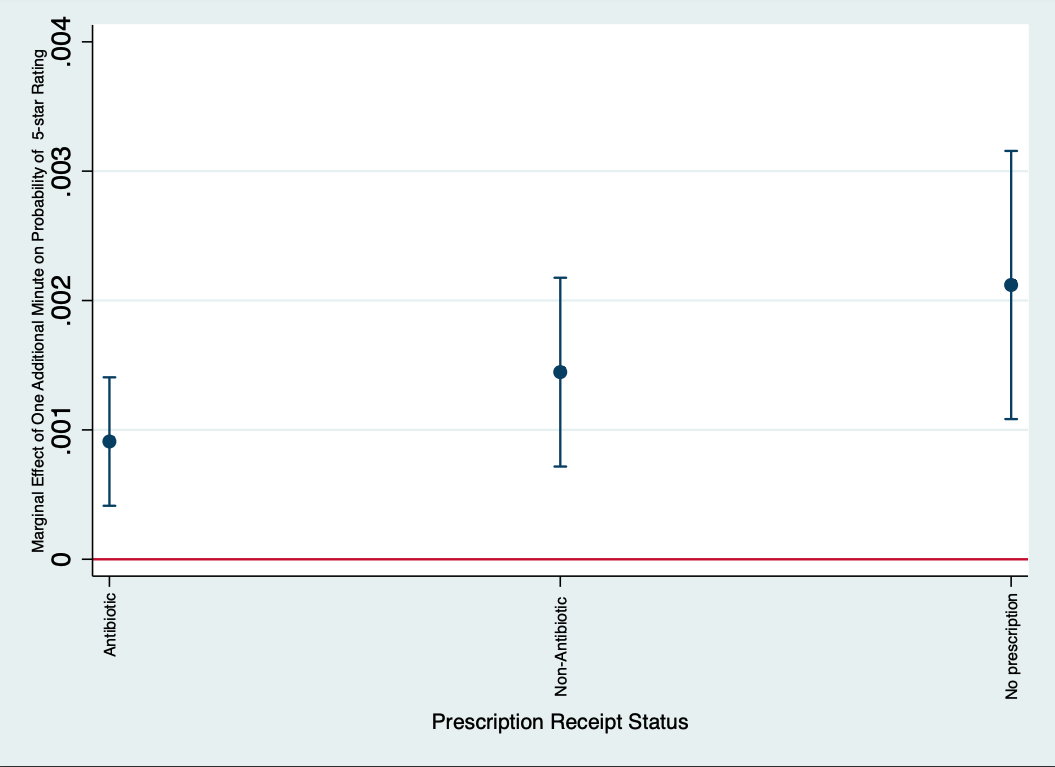


^a^Figure shows the marginal effect of one additional minute in visit duration on the adjusted probability of a 5-star rating, based on a multivariable logistic generalized estimating equations (GEE) with provider clustering, controlling for all variables as specified in the methods section, as well as an interaction term between duration of visit in minutes and prescription receipt category. STATA removed all observations with any missing values from the multivariable logistic regression (n=428). The effect is shown separately by prescription receipt. The dots represent the predicted marginal effect and the bars are the 95% confidence intervals. The marginal effect of one additional minute in visit duration for each prescription receipt status was statistically significant on the probability of a 5-star rating; however, the predicted marginal effect was not statistically significantly different between the three groups of prescription receipt.
